# Protective Role of Coenzyme Q10 in Acute Sepsis-Induced Liver Injury in BALB/c Mice

**DOI:** 10.1155/2020/7598375

**Published:** 2020-12-18

**Authors:** Qian-wei Li, Qin Yang, Hong-Yang Liu, Yu-ling Wu, Yu-Hua Hao, Xiao-Qing Zhang

**Affiliations:** ^1^Department of Gastroenterology, Affiliated Zhongshan Hospital of Dalian University, No. 6 Jiefang Street, Dalian, China; ^2^Department of Internal Medicine, Affiliated Zhongshan Hospital of Dalian University, No. 6 Jiefang Street, Dalian, China; ^3^Department of Heart Intensive Care Unit, The First Affiliated Hospital of Dalian Medical University, No. 193 Lianhe Road, Dalian, China; ^4^Department of Infection, Affiliated Zhongshan Hospital of Dalian University, No. 6 Jiefang Street, Dalian, China

## Abstract

Sepsis increases the risk of the liver injury development. According to the research works, coenzyme Q10 exhibits hepatoprotective properties in vivo as well as in vitro. Current work aimed at investigating the protective impacts of coenzyme Q10 against liver injury in septic BALB/c mice. The male BALB/c mice were randomly segregated into 4 groups: the control group, the coenzyme Q10 treatment group, the puncture and cecal ligation group, and the coenzyme Q10+cecal ligation and puncture group. Cecal ligation and puncture was conducted after gavagaging the mice with coenzyme Q10 during two weeks. Following 48 h postcecal ligation and puncture, we estimated hepatic biochemical parameters and histopathological changes in hepatic tissue. We evaluated the expression of factors associated with autophagy, pyroptosis, and inflammation. Findings indicated that coenzyme Q10 decreased the plasma levels in alkaline phosphatase, alanine aminotransferase, and aspartate aminotransferase in the cecal ligation and puncture group. Coenzyme Q10 significantly inhibited the elevation of sequestosome-1, interleukin-1*β*, oligomerization domain-like receptor 3 and nucleotide-binding, interleukin-6, and tumor necrosis factor-*α* expression levels; coenzyme Q10 also increased beclin 1 levels. Coenzyme Q10 might be a significant agent in the treatment of liver injury induced by sepsis.

## 1. Introduction

Sepsis is a lethal health issue worldwide, which has high incidence annually [1]. It is an organ dysfunction that is induced by a dysregulated host reaction to pathogen infection [2]. The mortality of sepsis is directly related to the development of organ dysfunction, but it is still not fully understood [3]. As the metabolic center of the body and a pivotal organ of the immune system, the liver is a primary organ in the response to sepsis and critically contributes in the sepsis pathogenesis [4]. Furthermore, recent advancements have reported the intimate interaction between cecal ligation and puncture- (CLP-) induced septic organ failure and cell dysfunction owing to autophagy and pyroptosis [5–7]. Additionally, multiple studies have indicated that autophagy and pyroptosis play important roles in liver physiology and pathology [7, 8].

Autophagy is a process that degrades damaged or abnormal cells and recycles the redundant or inefficient components to maintain cellular homeostasis [9, 10]. This involves a complex interplay of critical autophagic proteins, including beclin 1 and sequestosome-1 (p62). Pyroptosis can be described as certain programmed cell death, and the inflammatory cytokines release its characterization. It is also an inflammasome-activated process. The nucleotide-binding and oligomerization domain-like receptor 3 (NLRP 3) inflammasome is a molecular platform activated by signs of cellular danger through the secretion and maturation of proinflammatory cytokines like IL-18 and interleukin- (IL-) 1*β* [11, 12]. The NLRP 3 inflammasome crucially contributes in liver diseases, including ischemia/reperfusion injury, drug-induced hepatotoxicity, and fibrosis [13–15].

Despite extensive use of the “Surviving Sepsis” campaign guidelines, which are responsible for a worldwide decrease in mortality [16, 17], sepsis remains a leading cause of death [18]. Recently, various clinical experiments have suggested that novel strategies that focus on cell survival might be highly promising in improving clinical outcomes in septic patients [19]. Therefore, further investigation is essential to develop novel therapeutic interventions for the prevention of liver injury during sepsis.

Coenzyme Q10 (CoQ10) serves as an important electron carrier in the mitochondrial respiratory chain, and it is a natural lipophilic compound constructed in the mitochondrial inner membrane [20]. Previous studies have reported that CoQ10 can prevent the start and diffusion of lipid peroxidation, scavenge free radicals, and decrease proinflammatory cytokine production [21, 22] The deficiency of CoQ10 induced by mitochondrial failure in sepsis may play role in hypoxia, oxidative organ damage, hypoperfusion, and ultimately leading to death. A previous study revealed that CQ10 preconditioning enhanced autophagy and caused improvement in the cardiac function in rats having acute ischemia-reperfusion injury [23]. In addition, emerging research has demonstrated that CoQ10 attenuates NLRP 3 inflammasome activation and IL-1*β* serum levels in multiple acyl-CoA dehydrogenase deficiency (MADD) and fibromyalgia (FM) patients [24, 25].

However, the CoQ10 impact on septic liver injury induced by CLP is not still clear. The current work aims at investigating the effects of CoQ10 on autophagy, pyroptosis, and inflammatory injuries and the histopathological and biochemical alterations in the septic liver injury induced by CLP.

## 2. Material and Methods

### 2.1. Maintenance of Animal Models

Male BALB/c mice were prepared in Beijing. The mice samples were kept under stable situations, i.e., temperature of 23-25°C, 40-60% humidity, and a cycle of 12 h light and dark.

### 2.2. Development of a Murine Model with Sepsis

For induction of polymicrobial sepsis, a murine model was developed by performing CLP using an established method [26]. The mice samples undergone anesthesia with sodium pentobarbital (50 mg/kg, intraperitoneal injection). Following opening the peritoneum by the surgery, and exposure to the bowel, two-thirds of the cecum was tied and cut with a needle of 21-gauge. In order to extrude a little of feces, the site of the perforation was mildly pressed. It was placed in the peritoneal cavity. In the end, the laparotomy site was sutured. The same procedure was applied on Sham-operated mice, including opening the peritoneum by the surgery and the bowel exposure. Nevertheless, needle perforation of the cecum and the ligation were not carried out. The male BALB/c mice (8-week old) (*n* = 48) were segregated into 4 groups randomly (*n* = 12/group): control, CoQ10 (100 mg/kg/day); Sigma-Aldrich, St. Louis, MO, USA), CLP, and CLP+CoQ10. In the CLP+CoQ10 group, CLP was performed following gavaging the mice with CoQ10 for two weeks. After 48 hours following CLP, all mice were anesthetized with a dose of pentobarbital (100 mg/kg, intraperitoneally), blood samples were taken from the mice inferior vena cava, then the mice were euthanized by cervical dislocation, and respiration and heartbeat stopped indicating the death of the mice. Blood samples were gathered in serum tubes and kept at -80°C until further use. The fixation of the liver tissues' coronal sections was done in 10% formalin and soaked in paraffin for histological examination. The remnants of liver tissues were flash-frozen in liquid nitrogen and used to perform immunoblotting analyses or mRNA. All the studies on the animals were conducted based on the provided guides and followed international guidelines. The ethical committee of the Zhongshan Hospital of Dalian University provided the approval for the research.

### 2.3. Serum Analysis

ELISA kits (Nanjing Jiancheng Bioengineering Institute, Nanjing, China) were used for estimating the serum concentrations of alanine aminotransferase (ALT), alkaline phosphatase (ALP), and aspartate aminotransferase (AST) based on the guides provided by the manufacturer.

### 2.4. Staining of Hematoxylin-Eosin (H&E)

10% buffered formalin solution was used for fixation of the liver tissues done for 30 min and soaked in paraffin. Sections (4 *μ*m) were serially cut to perform the morphometric analysis of atherosclerotic lesions. These sections were stained using H&E to perform histological analysis using a light microscope (Olympus, Tokyo, Japan) (magnification 40x). As previously described [27, 28], kidney damage scores were determined according to the extent of kidney injury by two blinded researchers. Scoring was primarily based on the presence or absence of hemorrhaging, tubular cell necrosis, tubular dilatation, and cytoplasmic vacuole formation. The grading system was scored as follows: 0, 0% damage (normal kidney); 1, 0-5% damage (minimal damage); 2, 5-25% damage (mild damage); 3, 25-75% damage (moderate damage); and 4, 75-100% damage (severe damage).

### 2.5. Morphological and Immunohistochemistry Analysis

Immunohistochemical analysis was conducted by the use of a histone simple stain kit based on the guides provided by the manufacturer. The sections embedded in paraffin were deparaffinized using xylene, and they were rehydrated in decreasing serial dilutions of ethanol washes. These sections underwent treatment with 3% H_2_O_2_ in methanol for 15 minutes for inactivation of endogenous peroxidases. Afterward, they were incubated at ambient temperature for one hour with primary antibodies against beclin 1 and p62. A microscope was used for observing the stained tissue sections (Olympus, Tokyo, Japan) (magnification 40x).

### 2.6. RNA Isolation and Real-Time Chain Reaction (RT-PCR)

Isolation of total RNA from the LTs was done by the use of ISOGEN reagent based on the protocol provided by the manufacturer. Using a first-strand cDNA synthesis kit (TransGen, Beijing, China), complementary DNA (cDNA) was made from total RNA based on the protocol provided by the manufacturer. The specific gene expressions were analyzed in terms of quantity by running RT-PCR by the use of fluorescent SYBR Green technology. For normalization of the relative target gene expression, *β*-actin cDNA was quantified and amplified in the preparations of cDNA.  Table 1 presents the primer sequences used in the current work.

### 2.7. Western Blotting Analysis

Radio immunoprecipitation assay buffer (P0013B; Beyotime, Shanghai, China) was used for the extraction of proteins from the liver tissue. Samples were resolved via SDS-PAGE on a 10% gel, and protein bands were delivered into polyvinylidene fluoride (PVDF) membranes. PVDF membranes were hindered in Tris-buffered saline with 0.1% Tween-20 containing 5% skimmed milk. The primary antibody diluent (P0023A; Beyotime) was used for incubation, and it was mildly stirred overnight at 4°C. Primary antibodies against p62 (rabbit anti-p62 antibody, 1 : 1000; Proteintech), IL-1*β* (rabbit anti-beclin 1 antibody, *β*-actin (anti-*β*-actin, 1 : 1000; Cell Signaling Technology), NLRP 3 (rabbit anti-beclin 1 antibody, 1 : 000; Proteintech), and beclin 1 (rabbit anti-beclin 1 antibody, 1 : 000; Proteintech, 1 : 000; Cell Signaling Technology) were utilized in this study. The secondary antibodies (anti-rabbit Ig-G, 1 : 1000) were utilized for incubation of the membranes for one hour. The analysis was conducted 3 times independently. The levels of protein were normalized using protein/*β*-actin ratios so that the differences of loading are minimized. The NIH ImageJ software was used for quantification of the relative signal intensity.

### 2.8. Statistical Analysis

The data were represented as the mean ± SEM. The data were statistically investigated by the SPSS software version 23.0. Intergroup variation was estimated by performing subsequent Tukey's test and ANOVA. *P* < 0.05 was regarded as the significance level.

## 3. Results

### 3.1. Metabolic Characterization

**Figure 1:**
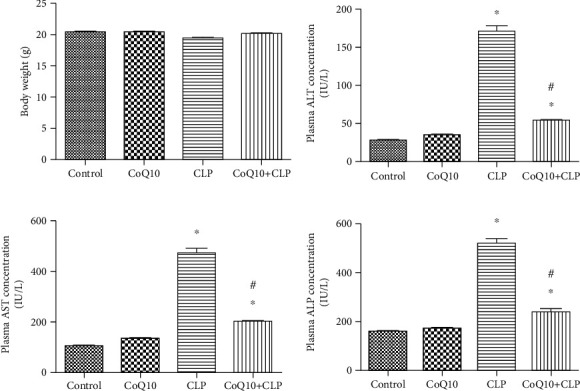
Metabolic data of the four groups of BALB/c mice after different treatments. Body weight and ALT, AST, and ALP levels are presented. Data are represented as mean ± SEM; *n* = 8 − 12 per group. ^∗^*P* < 0.05 vs. control group or CoQ10 group, ^#^*P* < 0.05 vs. CLP group.

Figure 1 summarizes the body weight and biochemical variables of the 4 groups of BALB/c mice with exposure to various treatments. The body weight did not show a difference in the groups. The CLP group exhibited a marked increase in the ALT, AST, and ALP levels; however, these levels were decreased in the CoQ10+CLP group.

### 3.2. CoQ10 Reduced Histopathological Injury in the Liver Tissues of the CLP Group

**Figure 2:**
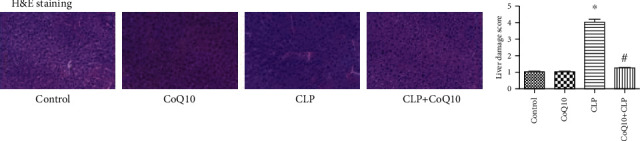
Histopathological damage in the liver tissues among the four groups of BALB/c mice subjected to different treatments. Data are represented as mean ± SEM; *n* = 3 per group. ^∗^*P* < 0.05 vs. control group or CoQ10 group, ^#^*P* < 0.05 vs. CLP group. Magnification 40x.

We performed H&E staining to evaluate the histopathological injury in the liver tissues (Figure 2) and found that the liver tissues in the control and CoQ10 groups showed normal hepatocytes with large spheroidal nuclei. In contrast, the CLP group exhibited marked hepatic strand disorganization, zonal necrosis, mononuclear cell infiltration, centrilobular swelling, and sinusoidal and centrilobular congestion, indicating liver injury. However, the administration of CoQ10 to mice prevented the degenerative changes in the hepatic structure induced by CLP.

### 3.3. CoQ10 Increased Beclin 1 and Decreased p62 Expression Levels in Liver of the CLP Group

**Figure 3:**
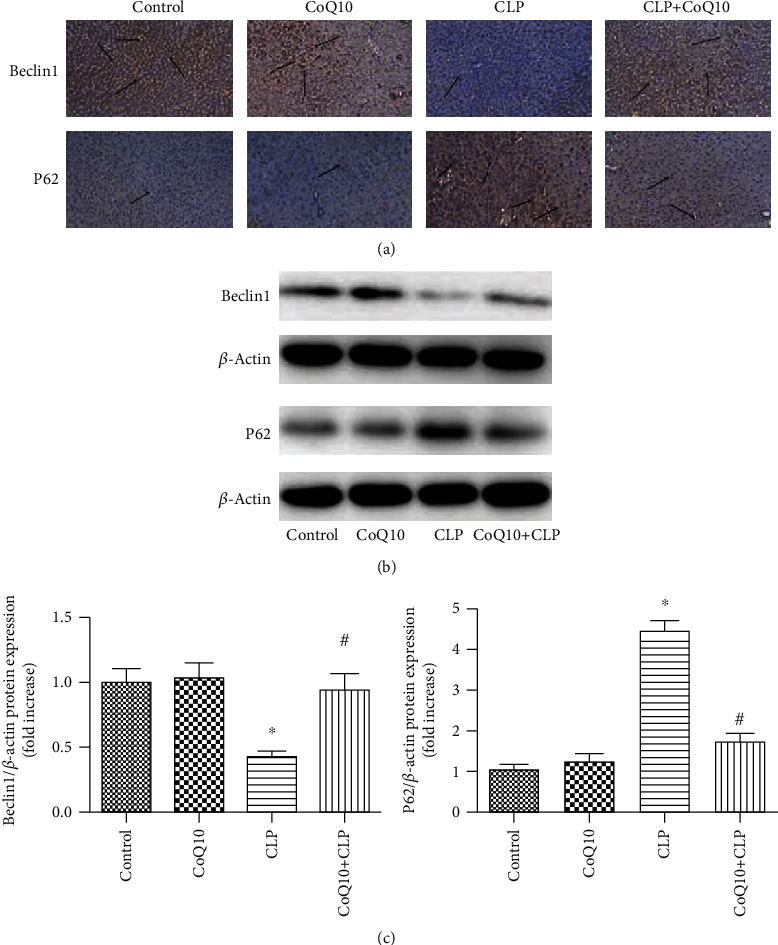
Beclin 1 and p62 expression levels in the liver among the four groups of BALB/c mice after different treatments. (a) Representative images of immunohistochemical analysis to detect the beclin 1 and p62 levels in the liver. Magnification 40x. Arrows indicate positively stained cells. (b) Immunoblotting to detect the beclin 1 and p62 expression levels in the liver. (c) Bar graph depicts the quantification of beclin 1 and p62 expression levels. Data are expressed as mean ± SEM; *n* = 3 − 4 in each group. ^∗^*P* < 0.05 vs. control group or CoQ10 group, ^#^*P* < 0.05 vs. CLP group.

Immunostaining was conducted for evaluation of beclin 1 and p62 expression levels in the liver (Figure 3(a)). The CoQ10+CLP group showed a remarkable rise in beclin 1 and reduction in p62 expression levels in the liver in comparison with those observed in the CLP group. Immunoblotting was conducted for beclin 1 and p62 (Figure 3(b)). We found that the expression level of beclin 1 showed a significant increase, but the expression level of p62 was considerably reduced in the CoQ10+CLP group in comparison with that of the CLP group (Figure 3(c)). According to the results, CoQ10 increased beclin 1 and reduced p62 expression levels in the CLP group.

### 3.4. CoQ10 Reduced NLRP 3 and IL-1*β* Expression Levels in Liver of the CLP Group

**Figure 4:**
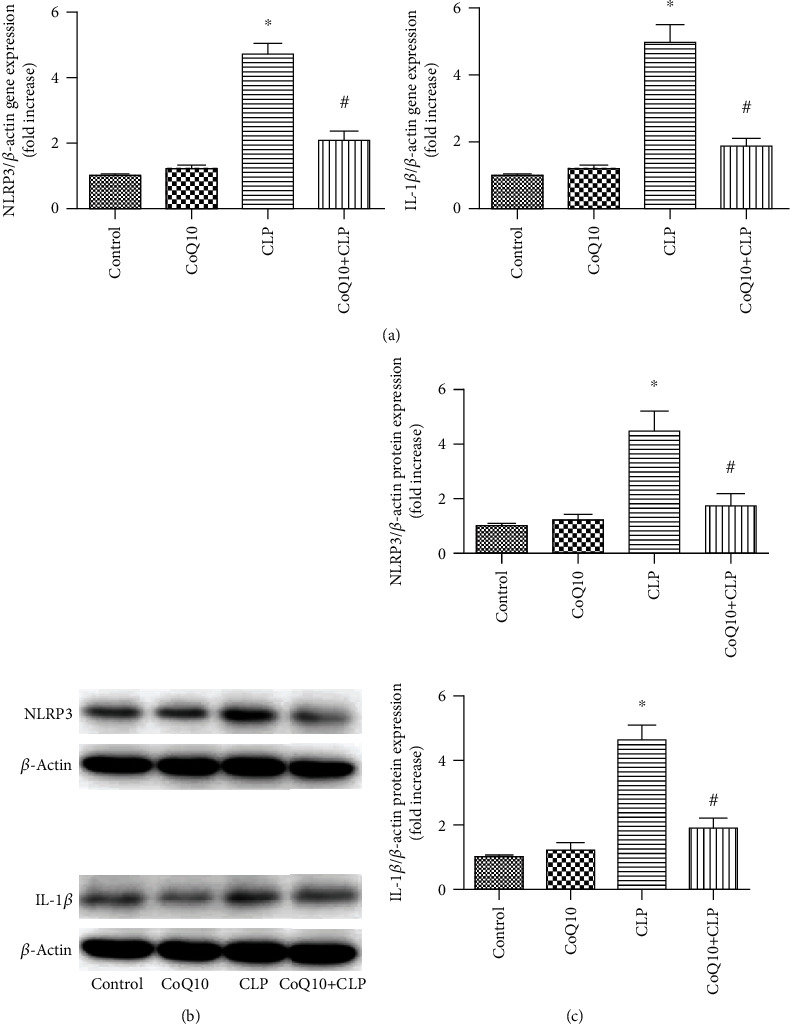
NLRP 3 and IL-1*β* mRNA and protein levels in the liver tissues of four groups of BALB/c mice after different treatments. (a) Relative mRNA expression levels of NLRP 3 and IL-1*β* in the liver tissues. (b) Immunoblotting to detect the NLRP 3 and IL-1*β* level in the liver tissues. (c) Bar graph depicts the quantification of NLRP 3 and IL-1*β* protein expression levels. Data are represented as mean ± SEM; *n* = 3 − 4 in each group. ^∗^*P* < 0.05 vs. control group or CoQ10 group, ^#^*P* < 0.05 vs. CLP group.

For studying the impacts of CoQ10 on the NLRP 3 and IL-1*β* regulation, we analyzed their gene expressions in liver obtained from mice taken from the 4 groups. The mRNA expression levels of NLRP 3 and IL-1*β* were upregulated in the CLP group. Nevertheless, this upregulation was undermined in the CoQ10+CLP group (Figure 4(a)). To verify the involvement of IL-1*β* and NLRP 3 protein levels, we performed immunoblotting in the respective treatment groups (Figure 4(b)). We found higher NLRP 3 and IL-1*β* protein expression levels in the CLP group; however, the CoQ10+CLP group showed significant suppression of NLRP 3 and IL-1*β* protein levels compared to the CLP group (Figure 4(c)).

### 3.5. CoQ10 Reduced IL-6 and Tumor Necrosis Factor- (TNF-) *α* Gene Expression Levels in the CLP Group Liver

**Figure 5:**
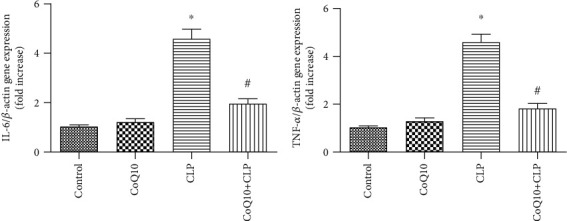
Expression of proinflammatory cytokines in liver obtained from the four groups of BALB/c mice after different treatments. Relative mRNA expression of IL-6 and TNF-*α* in the liver. Data are represented as mean ± SEM; *n* = 6 in each group. ^∗^*P* < 0.05 vs. control group or CoQ10 group, ^#^*P* < 0.05 vs. CLP group.

For investigating the contribution of proinflammatory cytokines in the gene expression level in mice liver, the TNF-*α* and IL-6 expressions were quantified by RT-PCR (Figure 5). In the CLP group, TNF-*α* and IL-6 expressions were upregulated. Nevertheless, this upregulation was undermined in the CoQ10+CLP group.

## 4. Discussion

It is well known that CoQ10 has an antioxidant effect due to its electron transfer ability; it has been given increasing attention due to its antioxidant and anti-inflammatory roles, especially in cardiac diseases and hypertension [29]. However, limited data exists about the effect of CoQ10 on CLP-induced septic liver injury. Therefore, we illustrated the effects of CoQ10 on CLP-induced septic liver injury using a murine model of CLP-induced polymicrobial sepsis.

In this study, there was not any important variation in body weight in four groups of samples. Like the findings by Lima GC et al., the serum concentrations of ALT, AST, and ALP in the CLP group were compared to concentrations in the control group [30]. Ozer et al. showed that elevations of serum AST and ALT induced by CLP were inhibited by CoQ10 [20]. Moreover, research works have indicated that CoQ10 treatment contributed to similar results regarding the serum AST, ALT, and ALP levels during thioacetamide- (TAA-) induced liver damage in rat models [31, 32]. In this study, the CoQ10 group displayed a significant reduction in these hepatic biochemical parameters compared to the CLP group. According to the histological evidence of liver injury as evaluated by performing H&E staining, pretreatment of murine models with CoQ10 completely protected mice against the liver damage caused by the CLP-induced sepsis.

Lin et al. indicated that notable autophagosomal accumulation was observed in the livers of septic patients as well as in experimentally induced sepsis [8]. Beclin 1 and p62 are two primary proteins contributed in autophagy. The ubiquitous expression of beclin 1 is one of the first mammalian autophagy effectors identified and crucially plays role in regulating both cell death and autophagy [23, 33]. We found that the level of beclin 1 in the liver was highly altered in the CLP group in comparison with the beclin 1 level in the control group. Similarly, pretreatment with CoQ10 before performing CLP increased the expression of beclin 1 in comparison with the CLP group. Liang et al. discovered that CoQ10 upregulated beclin 1 in acute myocardial ischemia-reperfusion injury [23]. Consistent with the findings of this research, other studies imply that the concentration of beclin 1 is generally correlated with autophagosomal activity [34, 35]. The multidomain adaptor protein p62 is homogeneously dispersed in the cell, and it contributed in many signal transduction pathways [8]. In this study, high levels of p62 aggregation in liver tissue were seen in the CLP group in comparison with the control group. Pretreatment with CoQ10 before performing CLP reduced p62 levels in comparison with the CLP group. A previous experiment showed that CoQ10 decreased p62 levels in acute myocardial ischemia-reperfusion injury [23]. Consistent with the findings of the current work, other studies imply that p62 concentration is generally inversely correlated with autophagosomal activity [36–38].

Generally, autophagy is a eukaryotic survival mechanism that recycles intracellular nutrients and maintains intracellular energy homeostasis [39]. In the present study, CoQ10 was hypothesized to prevent the septic liver injury induced by CLP via the enhancement of autophagy in vivo.

Pyroptosis is a novel, inflammatory form of programmed cell death. During sepsis, pyroptosis provides protection over host organisms against invasive bacterial infections to minimize tissue damage. Nevertheless, overactive pyroptosis can lead to multiple organ dysfunction syndrome (MODS), septic shock, or raised risk of secondary infection [7]. Currently, the leading mechanism of pyroptosis is speculated to be the NLRP 3 inflammasome: immune cells comprised of an NLRP 3 sensor, an apoptosis-associated speck-like protein containing (ASC) adaptor, and caspase-1, which is activated by pathogens [40]. Furthermore, the production of mature IL-1*β* depends on the formation of the NLRP 3 inflammasome [40].

Miao et al. and Ganz et al. observed that the protein expressions of IL-1*β* and NLRP 3 were the highest in an LPS-induced sepsis group in hepatic tissue [41]. Moreover, previous studies demonstrated that CLP-induced sepsis significantly increased IL-1*β* and NLRP 3 levels in the lung and kidney [42, 43]. Zhang et al. revealed that IL-1*β* and NLRP 3 were upregulated in alcoholic liver steatosis in vivo and in vitro [44]. In addition, El-Agamy et al. showed that lithocholic acid increased the expressions of NLRP 3 and IL-1*β* genes and proteins in hepatic tissue [45]. Recent research has demonstrated that CoQ10 attenuates NLRP 3 inflammasome activation and IL-1*β* serum levels in multiple acyl-CoA dehydrogenase deficiency (MADD) and fibromyalgia (FM) patients [24, 25]. Accordingly, in order to determine whether CoQ10 had any effect on the NLRP 3 inflammasome and IL-1*β* in CLP-induced septic liver injury, we measured the mRNA and protein levels of IL-1*β* and NLRP 3. We found higher NLRP 3 and IL-1*β* expression levels in the CLP group in comparison with those in the control group. Moreover, the CoQ10+CLP group showed a significant reduction of NLRP 3 and IL-1*β* levels in comparison with those in the CLP group. Our findings were gained using an animal model, and they indicated that the protective impact of CoQ10 against septic acute liver injury induced by CLP depends on inhibiting the protein and mRNA expressions of IL-1*β* and NLRP 3.

In this study, we demonstrated that mRNA levels of TNF-*α* and IL-6 in the liver were greatly upregulated in the CLP group in comparison with those in the control group. In the CoQ10 treatment condition, the expressions of TNF-*α* and IL-6 in the liver were suppressed as a response to CLP. A previous experimental study has indicated that CLP-induced elevations of inflammatory cytokine (TNF-*α* and IL-6) levels in blood were decreased by CoQ10 [20]. In graft versus host disease, the gene expressions of IL-6 and TNF-*α* reduced with CoQ10 treatment [46]. Additionally, a meta-analysis of RCTs suggests significant lowering effects of CoQ10 on TNF-*α* and IL-6 [47]. Therefore, we confirmed the anti-inflammatory roles of CoQ10 against CLP-induced septic acute liver injury.

Interestingly, in a double-blind, placebo, controlled randomized clinical trial, Sanoobar et al. confirmed that exogenous CoQ10 administration for 12 weeks could reduce IL-6 and TNF-*α* levels in patients with multiple sclerosis [48]. Donnino et al. showed that supplementation of exogenous Ubiquinol (the reduced form of CoQ10) could increase serum CoQ10 levels and improve mitochondrial function in patients with severe sepsis or septic shock in a pilot trial [49]. This provides a new therapeutic interventions for the early treatment of sepsis patients. Subsequently, in Soltani et al.'s research, they also showed that early administration of CoQ10 significantly reduced inflammatory markers in sepsis patients receiving Q10 and also reduced mortality in those patients [50]. However, the sample size is very small, and the clinical function of specific organs has not been evaluated, which needs further study.

## 5. Conclusions

In conclusion, the current study indicated that CoQ10 assists the reduction of septic liver injury as indicated by the upregulation of beclin 1 as well as the suppression of AST, ALT, ALP, p62, IL-6, TNF-*α*, NLRP 3, and IL-1*β*. These results offer novel information about the CoQ10 role in liver injury induced by sepsis and improve the potential of developing new therapeutic interventions to treat liver injury through autophagy and pyroptosis.

## Figures and Tables

**Table 1:** Primer oligonucleotide sequences.

Gene	Primers
TNF-*α*	F: 5′-TCTCATGCACCACCATCAAGGACT-3′ R: 5′-ACCACTCTCCCTTTGCAGAACTCA-3′
IL-6	F: 5′-TACCAGTTGCCTTCTTGGGACTGA-3′ R: 5′-TAAGCCTCCGACTTGTGAAGTGGT-3′
NLRP3	F: 5′-CTGCGGACTGTCCCATCAAT-3′ R: 5′-AGGTTGCAGAGCAGGTGCTT-3′
IL-1*β*	F: 5′-TGCCACCTTTTGACAGTGAT-3′ R: 5′-TGTGCTGCTGCGAGATTTGA-3′
*β*-Actin	F: 5′-CGATGCCCTGAGGGTCTTT-3′ R: 5′-TGGATGCCACAGGATTCCAT-3′

TNF-*α*: tumor necrosis factor-*α*; IL-6: interleukin-6; IL-1*β*: interleukin-1*β*.

## Data Availability

All datasets are available from the corresponding author upon reasonable request.

## Additional Points

*Certificate.* This document demonstrates that the English language has been thoroughly reviewed and is in compliance with academic publication standards.
